# Supplementary material for: Lateral membrane organization as target of an antimicrobial peptidomimetic compound
Source: Nat Commun. 2023 Jul 7;14:4038. doi: 10.1038/s41467-023-39726-5 (PMC10328936; doi:10.1038/s41467-023-39726-5)
Supplement: Supplementary file 1 — Supplementary Information [file 41467_2023_39726_MOESM1_ESM.pdf]

Supplementary Information

Lateral membrane organization as target of an antimicrobial peptidomimetic compound

Adéla Melcrová[1], Sourav Maity[1], Josef Melcr[2], Niels A.W. de Kok[3], Mariella Gabler[1], Jonne van der Eyden[1], Wenche Stensen[4], John S. M. Svendsen[4], Arnold J. M. Driessen[3], Siewert J. Marrink[1,2], Wouter H. Roos[1]

[1] Molecular Biophysics, Zernike institute for Advanced Materials, Rijksuniversiteit Groningen, the Netherlands
[2] Molecular Dynamics, Groningen Biomolecular Sciences & Biotechnology Institute, Rijksuniversiteit Groningen, the Netherlands
[3] Molecular Microbiology, Groningen Biomolecular Sciences & Biotechnology Institute, Rijksuniversiteit Groningen, the Netherlands
[4] Department of Chemistry, UiT Arctic University of Norway, Norway

Supplementary Note 1

Nano-indentations of *S. aureus* lipid membranes: Dissolution of lateral lipid domains leads to changes in membrane elasticity

We performed nano-indentation experiments[1–4] to evaluate the influence of the domains dissolution on the mechanical properties of the membrane (Figure S5). The nano-indentation data were fitted with a modified Hertz model, which encounters for a solid support below the membrane and ~1 nm water layer (d_{WL}) between the membrane and the support[2]:

$$\mathbf{F} = \frac{16Y}{9} R^{\frac{1}{2}}(z - z_0)^{\frac{3}{2}}[1 + 0.884\chi + 0.781\chi^2 + 0.386\chi^3 + 0.0048\chi^4] \qquad \text{(S1)}$$

where *R* is the radius of the AFM tip (typically 10 nm in our experiment) and $\chi = (R \times (z - z_0))^{1/2} / (z_0 - d_{WL})$. Young's modulus of the membrane *Y* and the vertical distance of the first tip–membrane interaction z_0 are the fitting parameters.

We focused on changes induced by AMC-109 at concentrations when the domains are accumulated or dissolved but the membrane is not yet expanded (0.5–1 μg/ml). The untreated *S. aureus* lipid membranes feature a double peak distribution of its thickness (Figure S5c) indicating a difference in height of the domains and the surrounding membrane. Upon the accumulation of the lateral lipid domains at 0.5 μg/ml AMC-109 the height differences between the two are less distinct as the thickness distribution appears to be broad but unimodal. Upon increasing the AMC-109 concentration to 1 μg/ml, we are at the edge concentration, when in some of the membrane patches the domains are accumulated, and in others the domains are already dissolved. These membranes display an increase in its Young's modulus from ~7 MPa for untreated membranes to ~17 MPa in the presence of 1 μg/ml AMC-109 (inset Figure S5b and S5f). Hence, dissolution of the lateral lipid domains and mixing of their content (likely cardiolipins and glycolipids[5,6]) with the surrounding membrane leads to overall stiffening of the membrane. This observation is in accordance

with literature[7] which shows that cardiolipin in small concentration increases lipid lateral packing and also compressibility and mechanical moduli of membranes.

The penetration force depends on the tip radius and the membrane composition. A comprehensive comparison of the tip size and membrane composition on the penetration force was previously reported[8]. Here a large range of penetration forces is found, from ~100 pN to ~10 nN, dependent on tip size and membrane composition. Our tips are expected to have a radius of curvature below 10 nm [9] and we conclude that the measured penetration forces fall within the previously reported experimental variation as a function of among others tip size and membrane composition.

Supplementary Figures

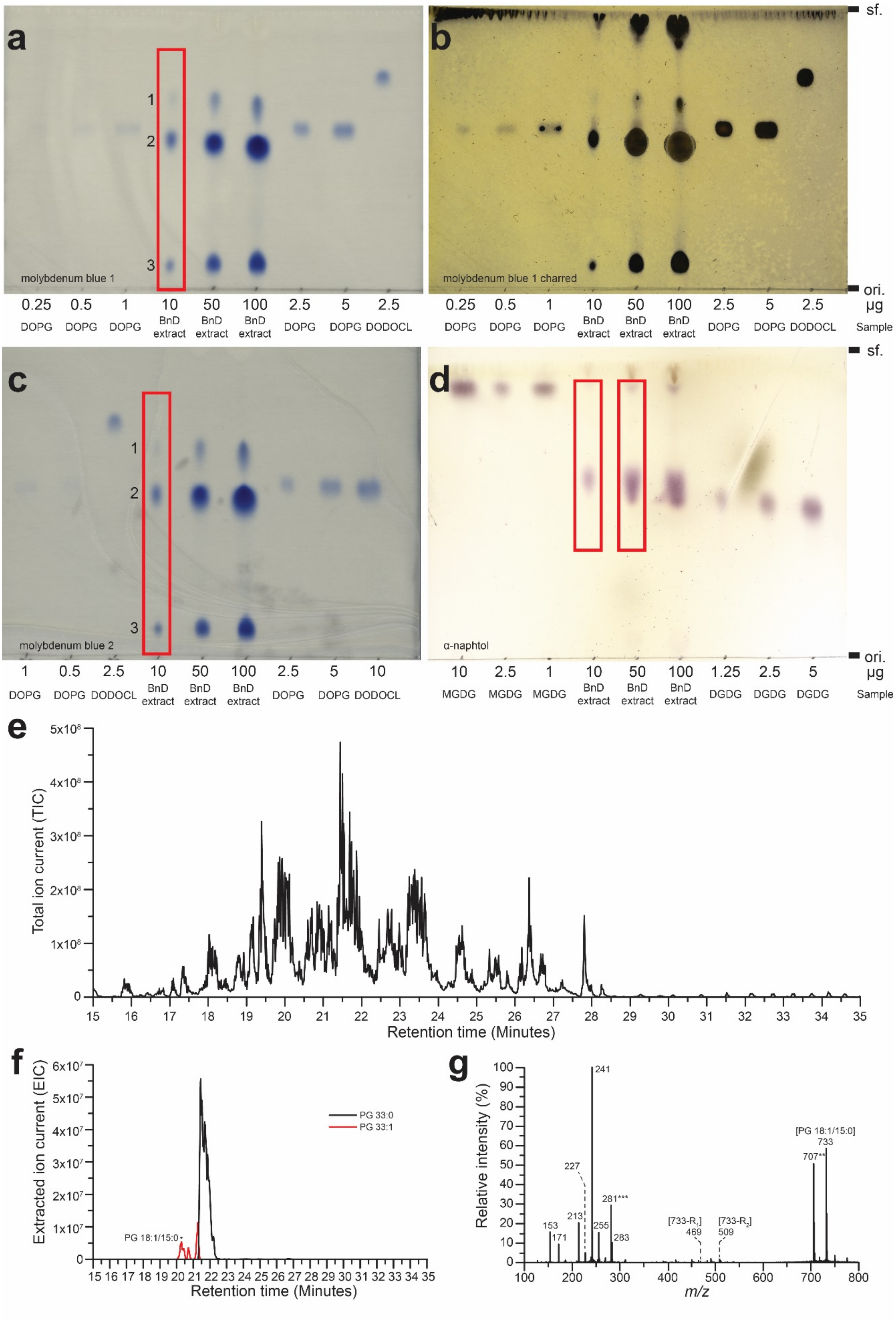

Fig. S1: Thin layer chromatography (TLC) analysis plates (**a–d**) and Mass Spectra (**e–g**) of the *S. aureus* lipid extract. **a** and **c** Molybdenum blue stains indicate the presence of phosphate groups as blue spots. **b** Upon strong heating of a molybdenum blue stained plate unsaturated compounds char and show up as black spots while the blue spots of saturated phospholipids blends away in the background. Even a small amount of the unsaturated acyl tails results in a dark spot. Hence, no quantitative information about the content of the unsaturated phospholipids can be made. **d** α-naphtol stain indicates the presence of carbohydrate groups as purple spots. Red rectangles indicate the area on the TLC plate analysed by densitometry (**a**, **c** and **d**). **e** Total ion current (TIC) spectrum of the *S. aureus* lipid extract. (**f**) Extracted ion count for PG 33:0 from the spectra shown in (**e**). The most abundant PG phospholipid (black), and the unsaturated species PG 33:1 (red) are shown. **g** Fragmentation spectrum of PG 33:1 (18:1/15:0) (taken at time annotated by * in panel **f**) is shown with the parent ion (mass 733) and two second order fragmentation products (mass 469 and 503) which were used to assign its regioisomeric identity are annotated in brackets. ** Annotates the parent ion of a co-eluting phospholipid, PG 31:0. *** Indicates an unsaturated fatty acid. Unsaturated PG phospholipids were responsible for ~10% of the total PG phospholipid signal. Source data for panels **e**, **f**, and **g** are provided as a Source Data file.

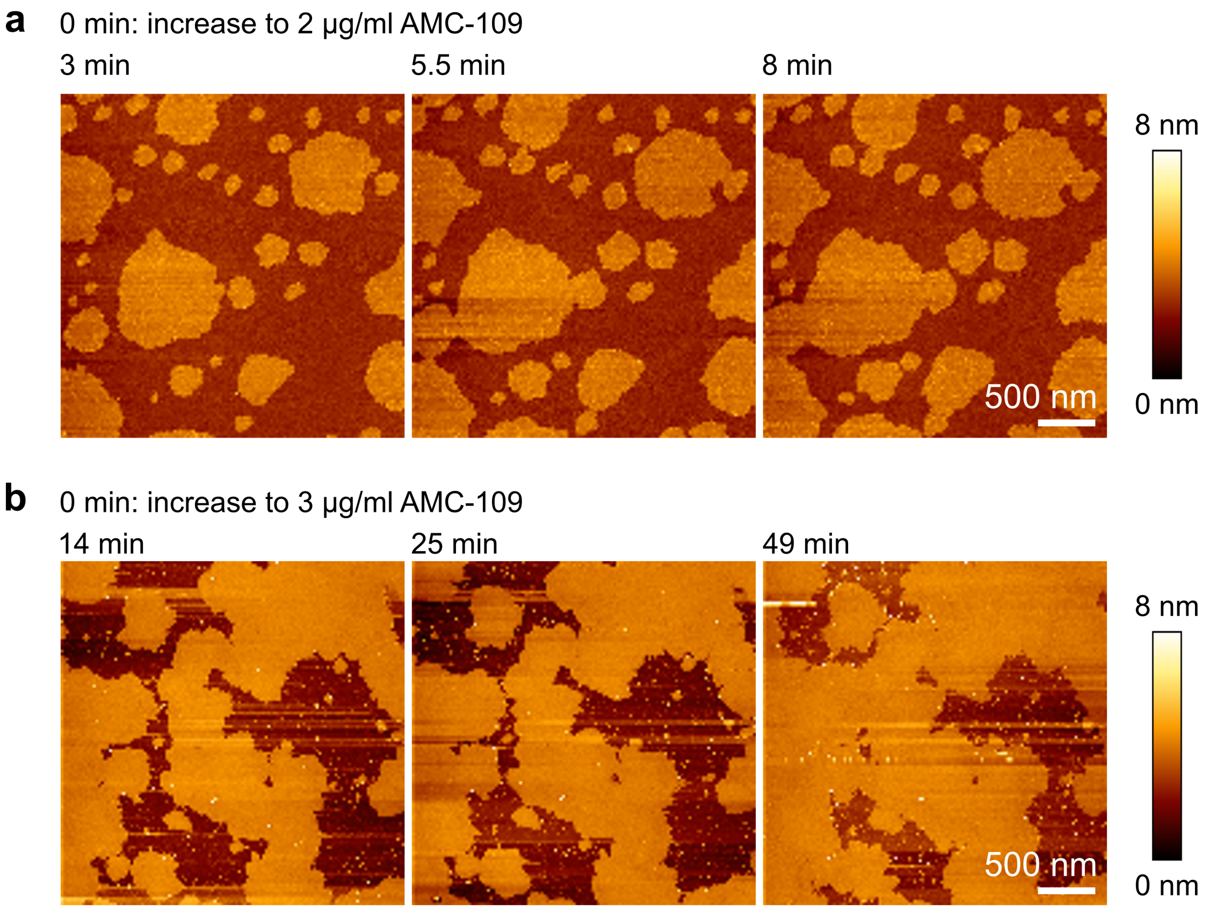

Fig. S2: Expansion of the *S. aureus* lipid membranes in time after the treatment with 2 (**a**) and 3 (**b**) µg/ml AMC-109. **a** 8 min after the addition of 2 µg/ml AMC-109 the system reaches equilibrium and the membrane stretching stops. In all measurements (N = 10) stretching stopped in <10 min. **b** At the concentration of 3 µg/ml, expansion is a continuous process and we see small changes even 49 minutes after the AMC-109 addition. All areas measured subsequently over the span of >40 min (N = 5) showed similar results.

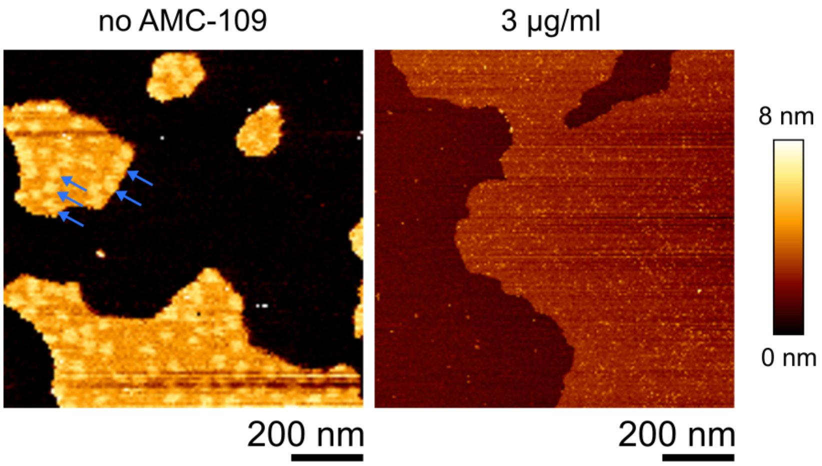

Fig. S3: 1x1 µm view of the changes in the *S. aureus* lipid membranes induced by the treatment with AMC-109. Blue arrows show lateral lipid domains in the untreated membrane. In the presence of 3 µg/ml AMC-109, i.e. above the minimal inhibitory concentration, the domains are lost, the membrane is expanded and thinned. The lighter color of the background indicates coverage of the mica surface with a layer of AMC-109 molecules. Similar results were observed in 5 independent measurements.

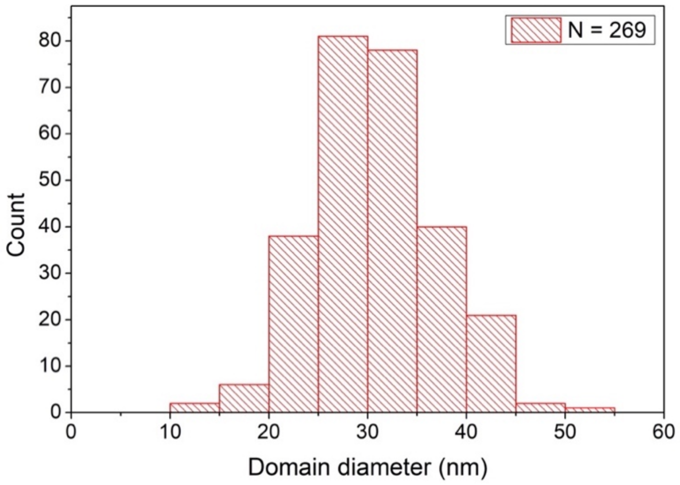

Fig. S4: Diameter of the lateral lipid domains in untreated *S. aureus* lipid membranes. Measured from HS-AFM images across four different experimental days. Average domain diameter is 30.9 ± 0.4 nm (N = 269). Source data for are provided as a Source Data file.

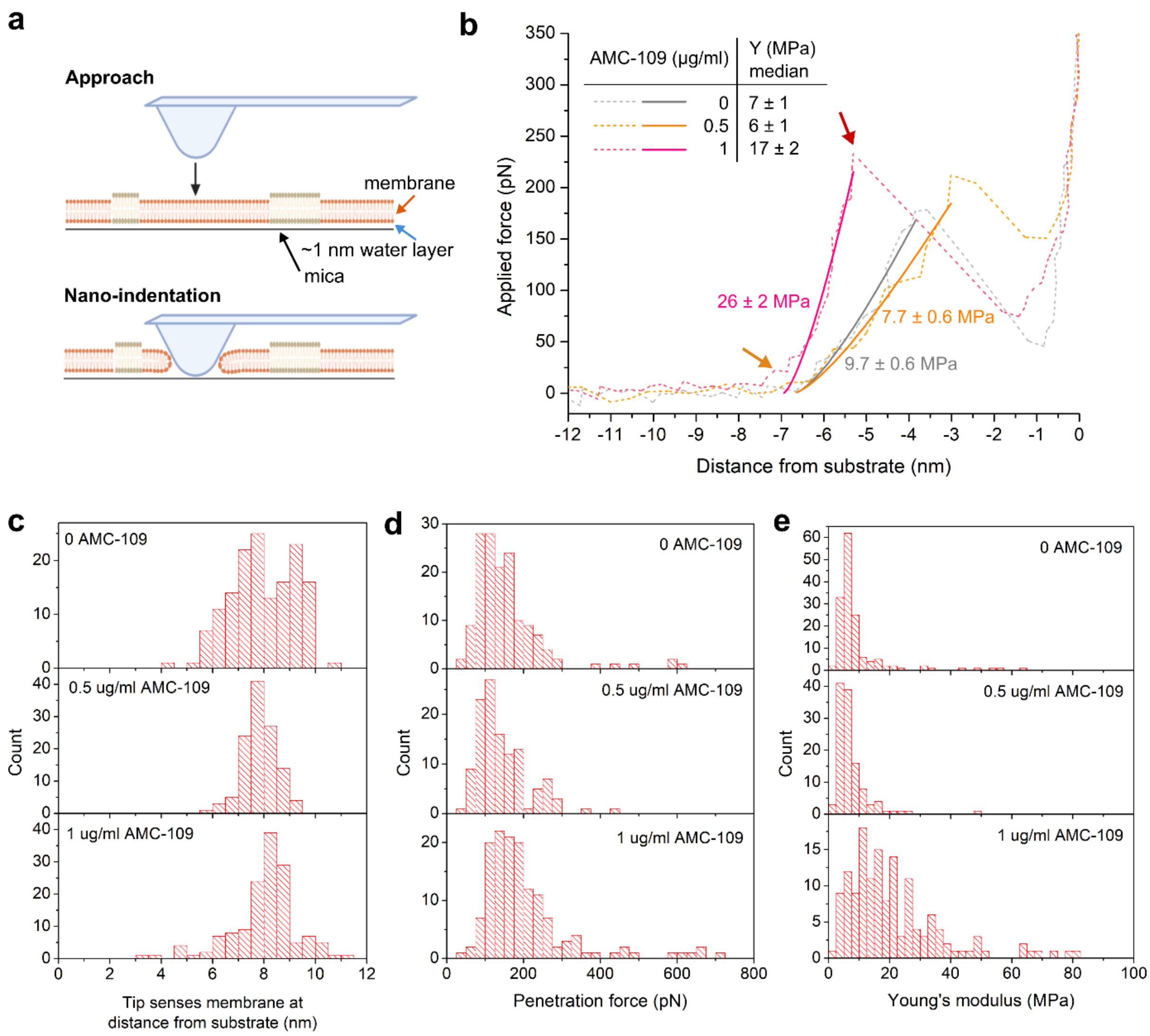

f

AMC-109	N total	Membrane height at tip interaction	Penetration force	Young's modulus
0	150	8 ± 1	130 ± 10 pN	7 ± 1 MPa
0.5 µg/ml	119	8 ± 1	120 ± 10 pN	6 ± 1 MPa
1 µg/ml	144	8 ± 1	170 ± 20 pN	17 ± 2 MPa

Fig. S5: Mechanical characterization of *S. aureus* lipid membranes upon the interaction with AMC-109. **a** Schematics of the nano-indentation experiment. Created with BioRender.com. AFM tip approaches the supported membrane (orange arrow), which lays on the solid mica substrate (black arrow) with ~1 nm layer of water[10,11] (blue arrow) in between the membrane and the support. **b** Representative force-indentation curves in the presence of 0 (grey), 0.5 (orange), and 1 µg/ml (pink) AMC-109. The orange arrow indicates the first contact between the AFM tip and the membrane. The red arrow shows the point, when the tip penetrates through the membrane. Solid curves are the fits of the region in between these two points using the modified Hertz model for supported thin layers Equation S1[2]. Inset table features the median values and standard errors of Young's moduli of *S. aureus* lipid membranes in the presence of 0, 0.5, and 1 µg/ml AMC-109 (N = 150, 119, and 144, respectively). **c** Distance from the substrate of the first interaction between the tip and the membrane. **d** Force needed to penetrate the membrane. **e** Young's modulus of the membranes. **f** Median values and standard errors of the respective parameters. Source data for panels **b**, **c**, **d**, and **e** are provided as a Source Data file.

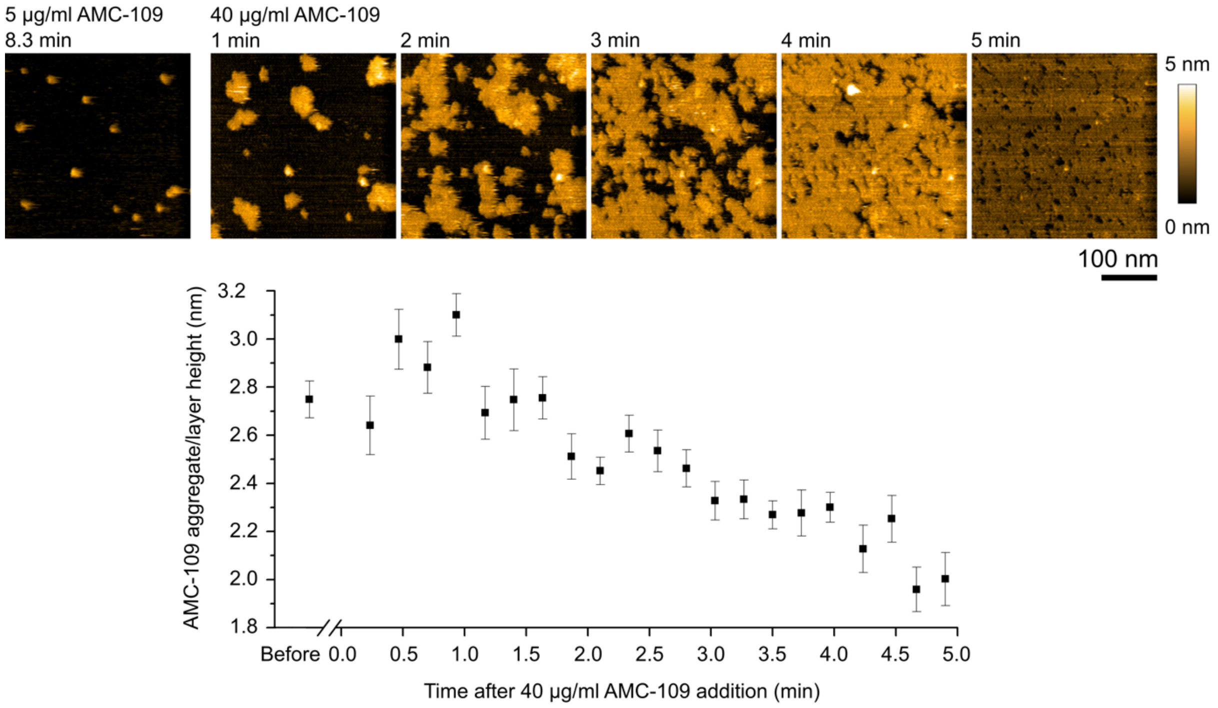

Fig. S6: AMC-109 on the mica background in HS-AFM imaging. In order to perform accurate thickness measurements of the lipid bilayer after addition of AMC-109 (Figure 2c), it is essential that the mica background does not get covered with AMC-109, as the membrane height is measured with respect to the mica height. Indeed, this is not the case for the used concentrations in Figure 2, as shown here: (Top left image) At 5 µg/ml AMC-109 individual aggregates attach on mica. The mica background can clearly be distinguished. (Top right images) Upon increasing the concentration of AMC-109 in the buffer to 40 µg/ml, more aggregates attach, gradually forming a uniform carpet-like layer. (Bottom) The height of the aggregates growing into the carpet-like layer decreases from 2.75 ± 0.08 nm (N = 104) for individual aggregates to 2.0 ± 0.1 nm (N = 25) at 294 s at a concentration of 40 µg/ml. Error bars represent standard error of the mean. Similar results were obtained in 2 independent experiments. Source data for panel **d** are provided as a Source Data file.

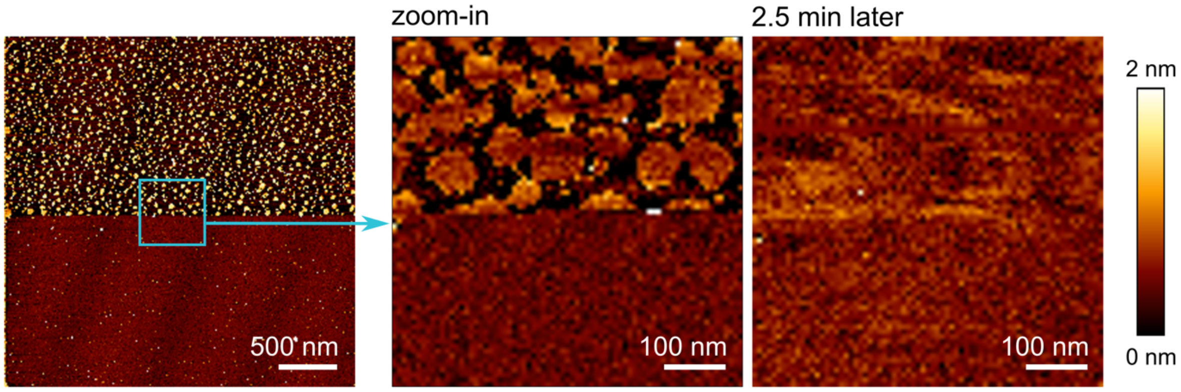

Fig. S7: AMC-109 on the mica background in AFM imaging. While during HS-AFM imaging no carpet-like layer of AMC-109 on the mica surface was formed during experiments at AMC-109 concentrations ≤ 5 µg/ml (Figure 2 and Figure S6), this is the case for AFM imaging. In particular in figure 1b it can be seen that the background height on the mica gradually increases after adding increasing amounts of AMC-109. A scratching experiment confirmed this. (Left) AFM imaging of a sample where 2 µg/ml AMC-109 was added to the PBS buffer onto a clean mica surface. The surface in the upper half of the left image was scratched in contact mode with 3 nN force. Next an image was taken of the scratched area (top) with the surrounding untouched layer (bottom) by AFM imaging in QI mode with 80 pN imaging force. The scratched material formed individual aggregates (bright yellow) attached on mica (black). Subsequent zoom-in image (middle) shows that the aggregates collapse again and reform the homogenous carpet-like layer. (Right) The carpet-like layer is restored within a few minutes. The height of the AMC-109 layer was estimated from the cross-sections through the AMC-109 layer and the adjacent mica surface as ~1 nm. The difference with HS-AFM imaging, where no carpet-like structure was observed for similar concentrations (Figure S6), is striking and is likely due to the longer experimental times and higher imaging force used at the AFM setup. Similar results were obtained for 3 independent scratching experiments.

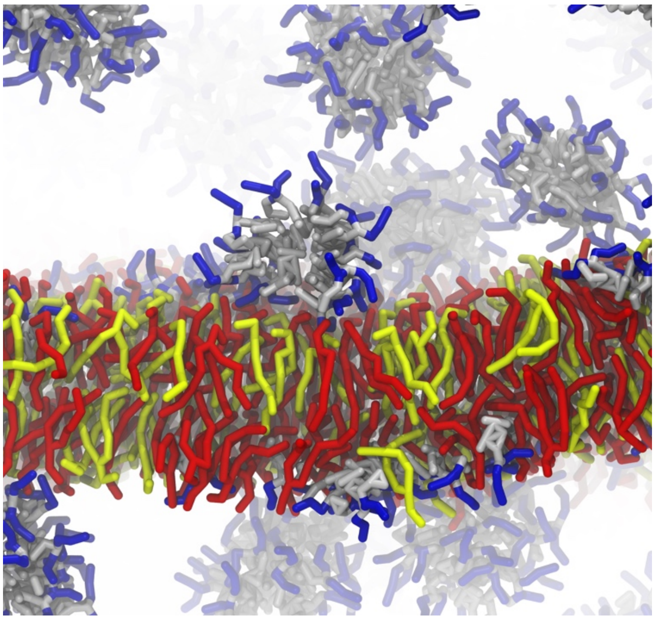

Fig. S8: Snapshot of the simulation box showing interaction of AMC-109 with the phospholipid membrane. Here, membrane consists of 50% POPC (yellow), 50% POPG (red). Multiple aggregates of AMC-109 (blue-grey) spontaneously formed in the bulk of water (white) and started attaching to the membrane from both sides.

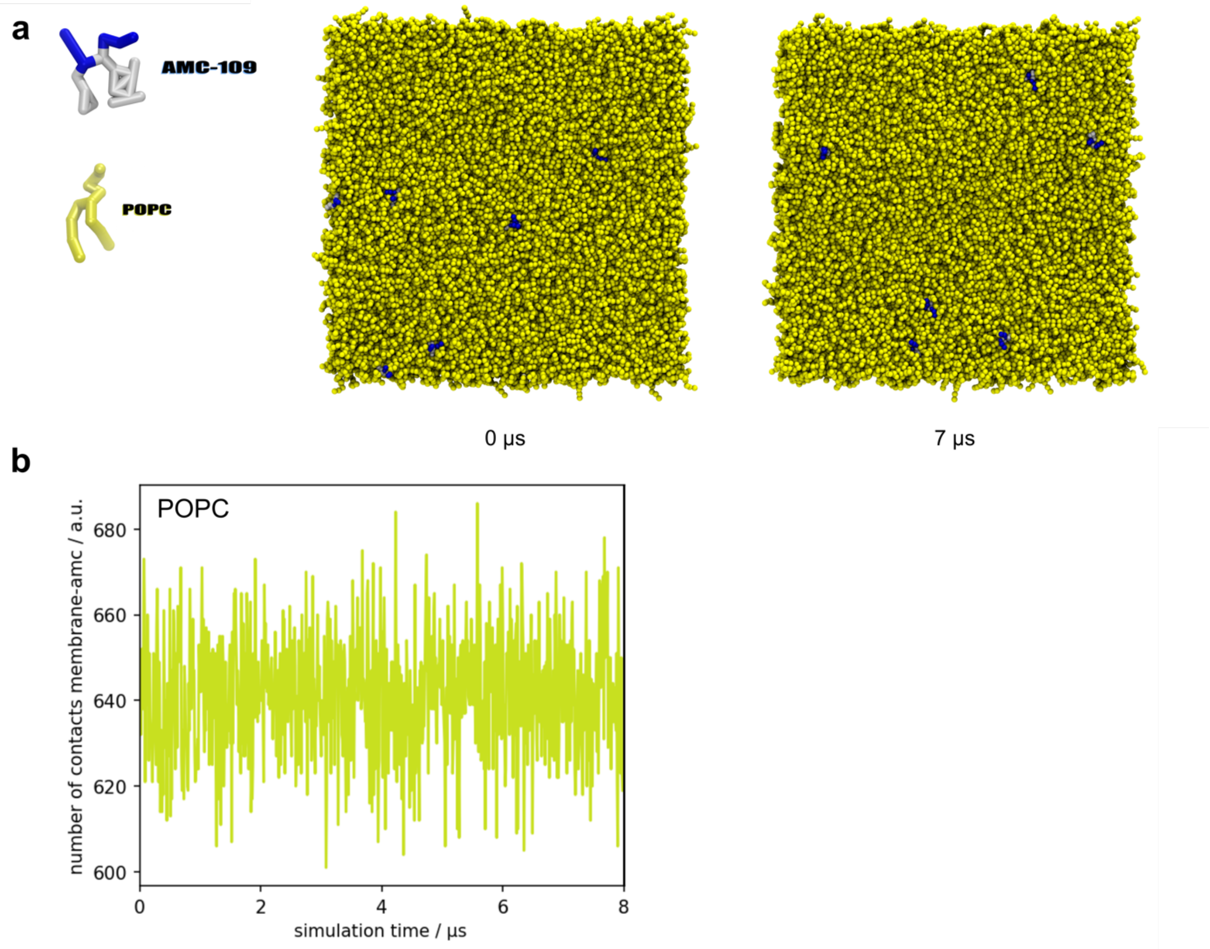

Fig. S9: AMC-109 monomers are stable in the neutral POPC membrane. In the beginning of the MD simulation, monomers of AMC-109 were added to the bulk water surrounding the POPC bilayer. The vast majority of the AMC-109 monomers formed aggregates as shown in Figure 3. However, 6 AMC-109 monomers encountered the POPC bilayer before entering any aggregate. This system was left equilibrating for 100 ns. Here, data from the 8 µs simulation following the equilibration are shown. **a** Top view of the POPC bilayer with the 6 AMC-109 monomers incorporated at the beginning of the simulation and after 7 µs. The monomers move laterally but do not change their position in the membrane or desorb back to the bulk water. **b** Time evolution of the number of contacts between the AMC-109 molecules and the POPC bilayer. Stable number of contacts again shows the stability of the AMC-109 monomers inside the membrane. Source data for panel **b** are provided as a Source Data file.

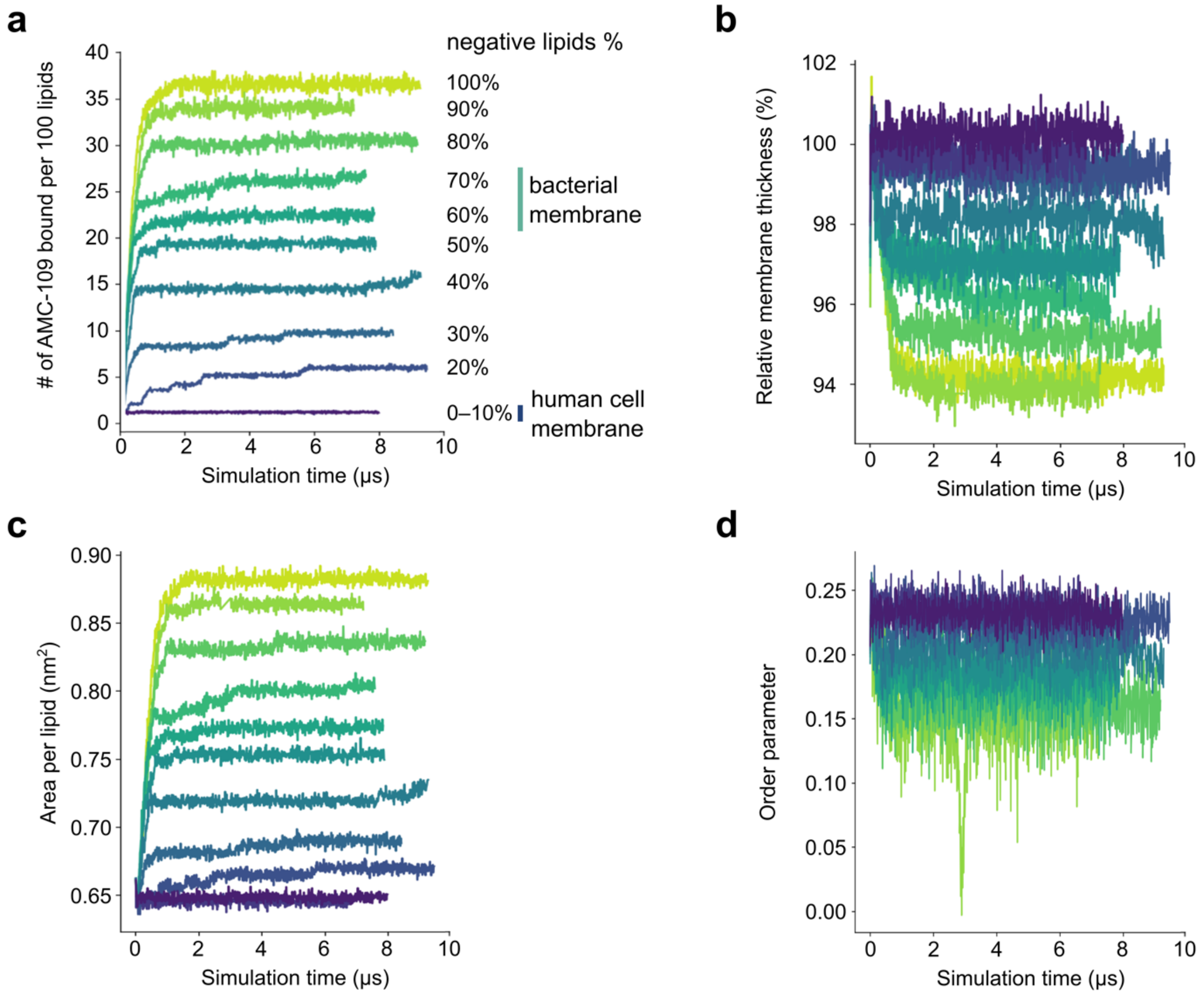

Fig. S10: MD simulations show membrane thinning, lipid lateral expansion, and change in lipid order upon the incorporation of AMC-109. **a** Number of contacts between AMC-109 and the simulated membranes composed of POPC lipids with varying content of POPG lipids from 0 to 100%. This panel is also shown in the main text as Figure 4a. **b** Relative membrane thickness in percent with respect to the initial value before AMC-109 insertion (counted as the average distance between the phosphate groups of the lipids in the opposite leaflets as implemented in FatSlim[12]). The higher content of the negatively charged lipids in the membrane results in higher AMC-109 insertion, which in turn leads to a decrease in the membrane thickness. **c** Area per lipid[12] (lateral area occupied by a single phospholipid) increases with AMC-109 adsorption leading to a lateral expansion of the membrane. The expansion is higher for higher amount of AMC-109 molecules inserted, i.e. for higher negative charge content. **d** POPC lipid tail order parameters decrease with the adsorbed amount of AMC-109. The order parameters were calculated for the bond orientation between the last two coarse grained particles of sn-1 tail of POPC lipids as described in [13]. Source data for all panels are provided as a Source Data file.

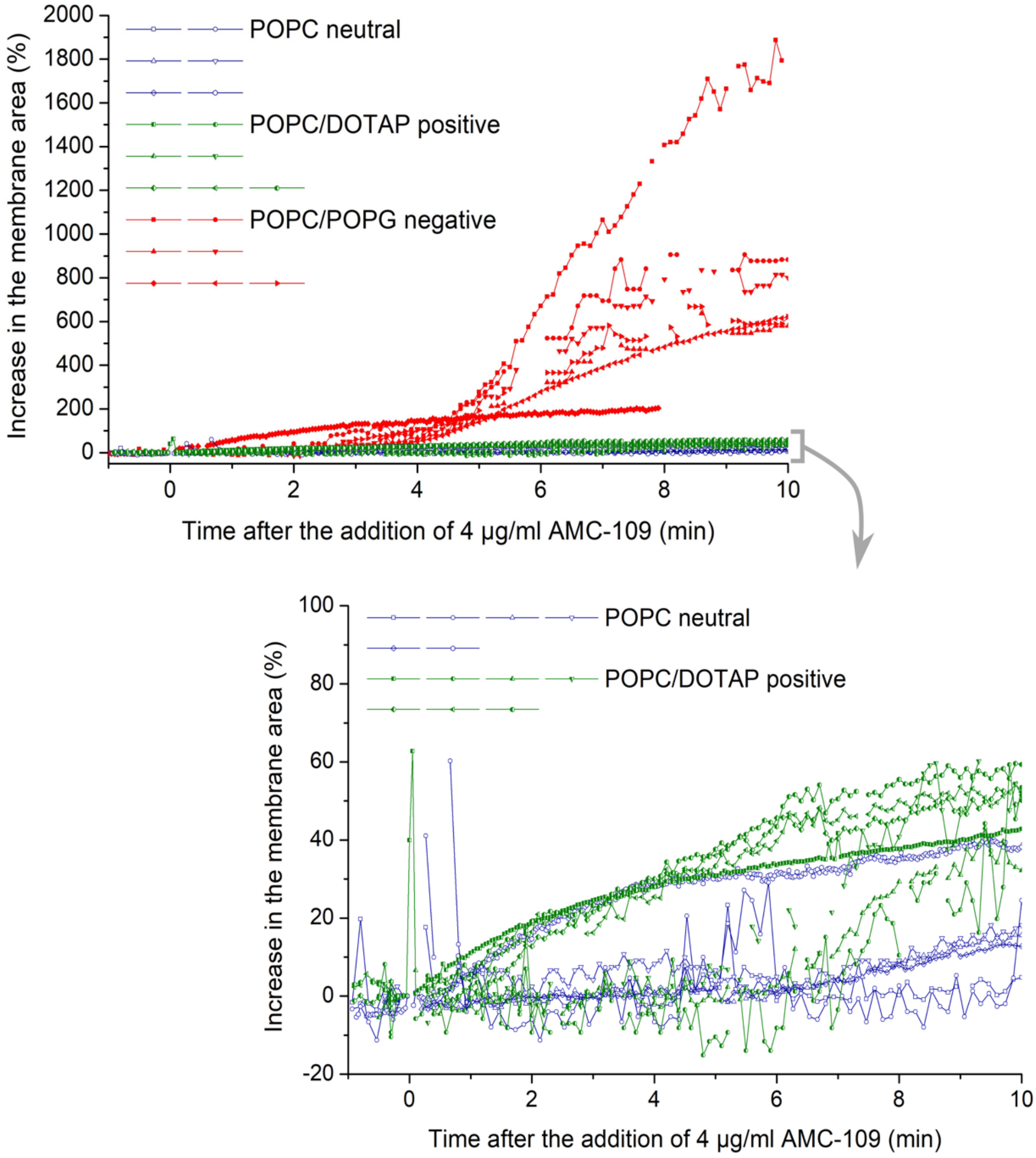

Fig. S11: Increase in the surface area of synthetic lipid membranes in time after treatment with 4 µg/ml AMC-109 analysed from the size of membrane patches visualized by HS-AFM. Growth curves for individual membrane patches, from which were calculated the mean values displayed at Figure 4d. Top panel shows the overview of all membrane compositions (POPC 100%, POPC/DOTAP 40/60 mol%, POPC/POPG 40/50 mol%). For good data visibility, a closer view on the growth curves of the neutral and positively charged membranes is shown at the lower panel. Data were acquired during 3 independent experiments for each membrane composition. Source data for are provided as a Source Data file.

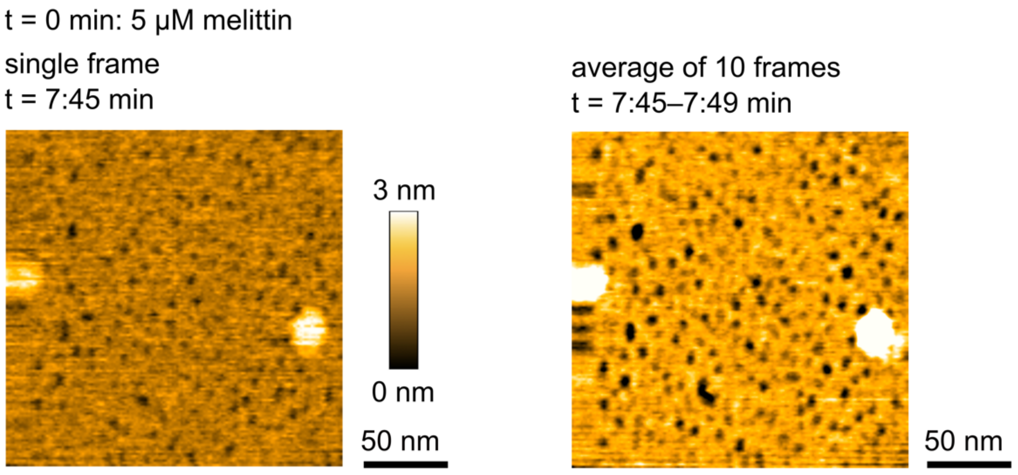

Fig. S12: Melittin induced pores in *S. aureus* lipid membrane visualized by HS-AFM after the addition of 5 µM melittin. (Left) Single frame from the HS-AFM video with the time step of 0.5 s. (Right) Superposition of 10 consecutive frames showing average intensity for each pixel from the superimposed frames. Individual pores in the membrane are visible as black dots and lateral domains as bright circles. Representative of 3 independent experiments is shown.

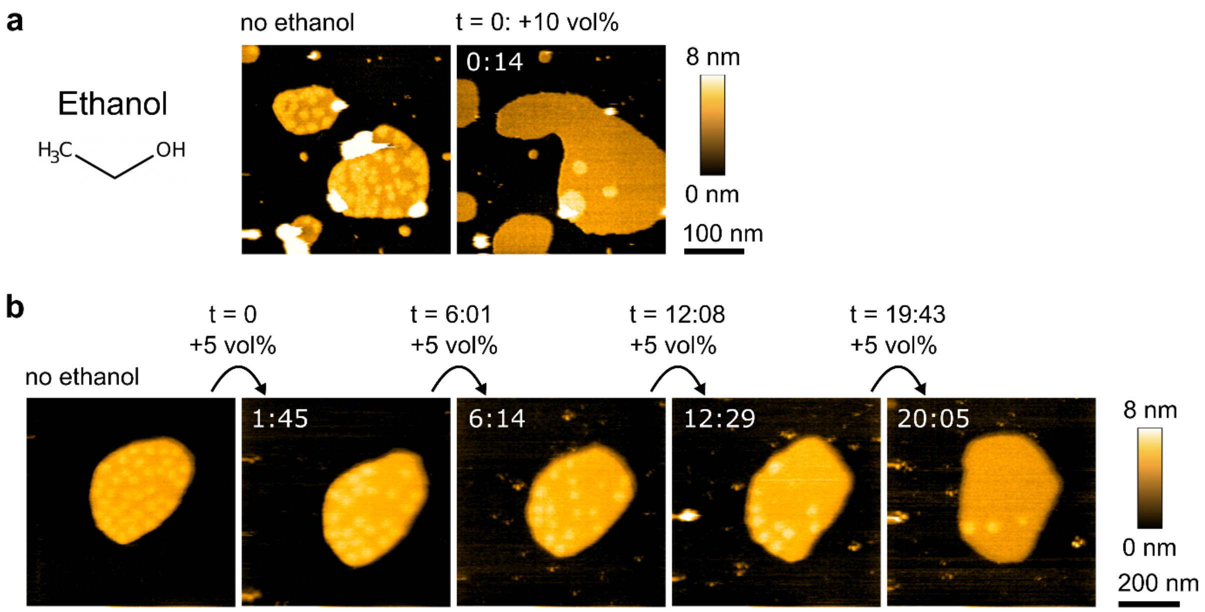

Fig. S13: Effects of ethanol on *S. aureus* lipid membranes observed by HS-AFM. **a** Chemical structure of ethanol and the membrane before and after the addition of 10 vol% of ethanol. We observe immediate domain dissolution and slight stretching of the membrane. **b** *S. aureus* lipid membrane before and after step by step addition of 5 vol% of ethanol. In order to observe the changes in more detail than in panel **a**, we decreased the amount of ethanol added in each step for this experiment. Some of the domains are dissolved within a few seconds after each addition of 5 vol% of ethanol. Slight stretching of the membrane is also observed. Similar results were observed in 5 independent AFM or HS-AFM experiments. Data from 2 independent HS-AFM experiments are visualized in panels **a** and **b**.

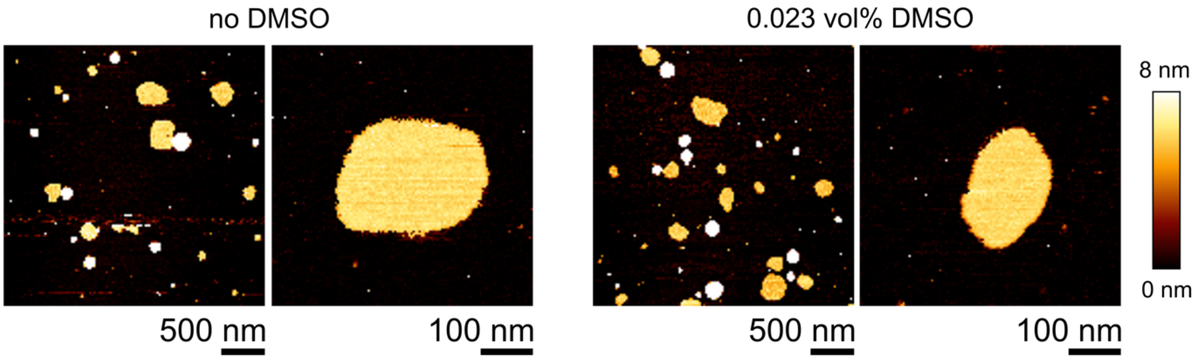

Fig. S14: Dimethyl sulfoxide (DMSO) at a concentration of 0.023 vol% does not affect the *S. aureus* lipid membranes. AFM images of *S. aureus* lipid membranes supported on mica in pure PBS buffer (left) and after the addition of 0.023 vol% of DMSO. The membranes do not show any sign of disruption. In the melittin experiments reported in Figure 5d and Figure S9, a residual concentration of respectively 0.010 and 0.015 vol% DMSO is present. This test experiment was performed once.

Supplementary References:

1. Roos, W. H., Bruinsma, R. & Wuite, G. J. L. Physical virology. *Nat Phys* **6**, 733–743 (2010).

2. Stetter, F. W. S. & Hugel, T. The nanomechanical properties of lipid membranes are significantly influenced by the presence of ethanol. *Biophys J* **104**, 1049–1055 (2013).

3. Calò, A. *et al.* Force measurements on natural membrane nanovesicles reveal a composition-independent, high Young's modulus. *Nanoscale* **6**, 2275–2285 (2014).

4. Garcia-Manyes, S., Oncins, G. & Sanz, F. Effect of ion-binding and chemical phospholipid structure on the nanomechanics of lipid bilayers studied by force spectroscopy. *Biophys J* **89**, 1812–1826 (2005).

5. Lopez, D. Molecular composition of functional microdomains in bacterial membranes. *Chem Phys Lipids* **192**, 3–11 (2015).

6. García-Fernández, E. *et al.* Membrane Microdomain Disassembly Inhibits MRSA Antibiotic Resistance. *Cell* **171**, 1354-1367.e20 (2017).

7. Wilson, B. A., Ramanathan, A. & Lopez, C. F. Cardiolipin-Dependent Properties of Model Mitochondrial Membranes from Molecular Simulations. *Biophys J* **117**, 429–444 (2019).

8. Saavedra V., O., Fernandes, T. F. D., Milhiet, P. E. & Costa, L. Compression, Rupture, and Puncture of Model Membranes at the Molecular Scale. *Langmuir* **36**, 5709–5716 (2020).

9. Majewska, M., Mrdenovic, D., Pieta, I. S., Nowakowski, R. & Pieta, P. Nanomechanical characterization of single phospholipid bilayer in ripple phase with PF-QNM AFM. *Biochim Biophys Acta Biomembr* **1862**, 183347 (2020).

10. Kim, J., Kim, G. & Cremer, P. S. Investigations of water structure at the solid/liquid interface in the presence of supported lipid bilayers by vibrational sum frequency spectroscopy. *Langmuir* **17**, 7255–7260 (2001).

11. Su, Z., Juhaniewicz-Debinska, J., Sek, S. & Lipkowski, J. Water Structure in the Submembrane Region of a Floating Lipid Bilayer: The Effect of an Ion Channel Formation and the Channel Blocker. *Langmuir* **36**, 409–418 (2020).

12. Buchoux, S. FATSLiM: a fast and robust software to analyze MD simulations of membranes. *Bioinformatics* **33**, 133–134 (2017).

13. Melcr, J., Ferreira, T. M., Jungwirth, P. & Ollila, O. H. S. Improved Cation Binding to Lipid Bilayers with Negatively Charged POPS by Effective Inclusion of Electronic Polarization. *J Chem Theory Comput* **16**, 738–748 (2020).
