# Supplementary material for: Lateral membrane organization as target of an antimicrobial peptidomimetic compound
Source: Nat Commun. 2023 Jul 7;14:4038. doi: 10.1038/s41467-023-39726-5 (PMC10328936; doi:10.1038/s41467-023-39726-5)
Supplement: Supplementary file 3 — Description of Additional Supplementary Files [file 41467_2023_39726_MOESM3_ESM.pdf]

Description of Additional Supplementary Files:

Supplementary Movie 1: Dynamics of lateral domains in S. aureus lipid membranes, imaged by HS-AFM. HS-AFM movie with a time step between the frames of 0.3 s. The movie shows dynamic lateral movement of the domains in the S. aureus lipid membrane deposited on mica. Mica is depicted in black, membrane dark gold and the moving domains light yellow. The domains do not noticeably change their size or shape, and do not spontaneously merge. The dark blue dot highlights one of the domains and its diffusion.

Supplementary Movie 2: Dissolution of lateral domains and expansion of S. aureus lipid membranes after AMC-109 treatment, imaged by HS-AFM. HS-AFM movie showing how the membrane is affected by the AMC-109 addition. Membrane is depicted in dark gold, lateral domains in yellow, surrounding mica in black, and unruptured membrane vesicle in white. The movie follows the membrane right after treatment with 2 µg/ml AMC-109. The domains cluster together but do not fully merge, and gradually dissolve into the surrounding membrane. Increasing of the AMC109 concentration to 4 µg/ml leads to complete dissolution of the remaining domains and expansion and thinning of the membrane. Thinning can be concluded from the increasingly darker colour (and therefore lower height) of the membrane. Time step between the individual images is 2 s.

Supplementary Movie 3: Formation of a continuous layer of AMC-109 molecules on mica, imaged by HS-AFM. HS-AFM movie of AMC-109 micelles attaching to freshly cleaved mica surface. No membrane is present in this experiment. Mica is depicted in black, AMC-109 aggregates in yellow. Addition of 2 µg/ml AMC109 to the buffer on mica results in the attachment of individual globular shaped aggregates on the mica surface with a height of 2.75 ± 0.08 nm (height distribution in Figure 3e). After 8:40 min of the experiment, we increased the AMC-109 concentration to 4 µg/ml, which resulted in a further attachment of a large amount of micelles on the mica surface gradually forming an uniform AMC-109 layer with a thickness of ~2 nm. Time step between the individual images is 2 s.

Supplementary Movie 4: 1 µs MD trajectory showing the whole simulation box. The movie shows the first 1 µs of the AMC-109 micelles attachment to the POPG/POPC (60:40 mol%) membrane. Individual micelles attach to the membrane and gradually dissolve into it. After 1 µs the AMC-109 attachment to the membrane is saturated as indicated in Figure 4a. Notice that the membrane in the end of the movie is wider and thinner, which is in accordance with our experimental observations.

Supplementary Movie 5: AMC-109 micelle incorporation into the model membrane from MD simulations. The movie shows a single AMC-109 micelle interacting with the POPG/POPC (60:40 mol%) membrane. All the other micelles and water are hidden from the view in order to focus the viewer to the individual process. The micelle comes from the water phase, peripherally attaches to

the membrane, and gradually dissolves into the membrane inserting all the AMC-109 monomers in between the lipids. The whole movie covers 350 ns of the simulation.

Supplementary Movie 6: Dissolution of lateral domains and expansion of S. aureus lipid membranes after the treatment with the disinfectant BAK, imaged by HS-AFM. HS-AFM movie showing how the membrane is affected by the addition of the disinfectant BAK. The membrane is depicted in dark gold, lateral domains in yellow, surrounding mica in brown. At the start of the movie the membrane is untreated with the domains randomly distributed and moving. With the addition of 5 µg/ml BAK the domains cluster together, then dissolve into the membrane, which is followed by expansion of the membrane. Time step between the individual images is 0.5 s.
