# Supplementary material for: Lateral membrane organization as target of an antimicrobial peptidomimetic compound
Source: Nat Commun. 2023 Jul 7;14:4038. doi: 10.1038/s41467-023-39726-5 (PMC10328936; doi:10.1038/s41467-023-39726-5)
Supplement: Supplementary file 10 — Reporting Summary [file 41467_2023_39726_MOESM10_ESM.pdf]

nature portfolio

Corresponding author(s): Wouter H. Roos

Last updated by author(s): Jun 15, 2023

Reporting Summary

Nature Portfolio wishes to improve the reproducibility of the work that we publish. This form provides structure for consistency and transparency in reporting. For further information on Nature Portfolio policies, see our Editorial Policies and the Editorial Policy Checklist.

Statistics

For all statistical analyses, confirm that the following items are present in the figure legend, table legend, main text, or Methods section.

n/a	Confirmed	
☐	☒	The exact sample size (*n*) for each experimental group/condition, given as a discrete number and unit of measurement
☐	☒	A statement on whether measurements were taken from distinct samples or whether the same sample was measured repeatedly
☒	☐	The statistical test(s) used AND whether they are one- or two-sided<br>*Only common tests should be described solely by name; describe more complex techniques in the Methods section.*
☒	☐	A description of all covariates tested
☒	☐	A description of any assumptions or corrections, such as tests of normality and adjustment for multiple comparisons
☐	☒	A full description of the statistical parameters including central tendency (e.g. means) or other basic estimates (e.g. regression coefficient) AND variation (e.g. standard deviation) or associated estimates of uncertainty (e.g. confidence intervals)
☒	☐	For null hypothesis testing, the test statistic (e.g. *F*, *t*, *r*) with confidence intervals, effect sizes, degrees of freedom and *P* value noted<br>*Give P values as exact values whenever suitable.*
☒	☐	For Bayesian analysis, information on the choice of priors and Markov chain Monte Carlo settings
☒	☐	For hierarchical and complex designs, identification of the appropriate level for tests and full reporting of outcomes
☒	☐	Estimates of effect sizes (e.g. Cohen's *d*, Pearson's *r*), indicating how they were calculated

Our web collection on statistics for biologists contains articles on many of the points above.

Software and code

Policy information about availability of computer code

Data collection	AFM data were collected using the JPK Nanowizard Control Software v.6, version 6.1.202.<br>HS-AFM data were collected using Eagle 2.5.9 software developed in Kanazawa-University Biophysics Lab.<br>Fluorescence leakages were collected using the software of Fluorometer QuantaMaster 40.<br>MD simulations were set up using tool Insane (ref 81) and the GROMACS tool gmx solvate (ref. 82). Simulations were run using GROMACS.
Data analysis	Both the AFM images and the force curves were processed using JPK Data Processing Software, version 6.1.142. Exported force-curves were analyzed in OriginPro 8.5.0 SR1.<br>HS-AFM data were processed using Kodec4, Copyright 2006-2008 N. Kodera, M. Sakashita, M. Imai, Kanazawa-University Biophysics Lab.<br>ImageJ 1.53t was used for processing of HS-AFM movies.<br>Statistics data were handled in Microsoft Excel Pro Mac, version 16.72 and Microsoft Excel 2019.<br>MD simulations data were analyzed using GROMACS tool gmx midist, and scripts that are openly available at https://doi.org/10.5281/zenodo.7990762.<br>Figures were prepared using Inkscape 1.0rc2 and 0.92.

For manuscripts utilizing custom algorithms or software that are central to the research but not yet described in published literature, software must be made available to editors and reviewers. We strongly encourage code deposition in a community repository (e.g. GitHub). See the Nature Portfolio guidelines for submitting code & software for further information.

Data

Policy information about availability of data

All manuscripts must include a data availability statement. This statement should provide the following information, where applicable:

- Accession codes, unique identifiers, or web links for publicly available datasets
- A description of any restrictions on data availability
- For clinical datasets or third party data, please ensure that the statement adheres to our policy

The data that supports the findings of this study can be found in the manuscript, its Supplementary Information, and provided Source Data file. Unprocessed AFM images, force-distance curves used for mechanical characterization, and raw mass spectroscopy data are available in an open public repository (DataverseNL, Ref. 86). Scripts used to analyze the simulations, molecular topology of AMC-109 and simulation setup files in GROMACS format, and trajectories of the lipid bilayers with AMC-109 molecules are available in a public repository (Zenodo, Ref. 85). Unprocessed HS-AFM data and data from experimental repetitions are available from the corresponding author upon reasonable request.

Human research participants

Policy information about studies involving human research participants and Sex and Gender in Research.

Reporting on sex and gender	N/A
Population characteristics	N/A
Recruitment	N/A
Ethics oversight	N/A

Note that full information on the approval of the study protocol must also be provided in the manuscript.

Field-specific reporting

Please select the one below that is the best fit for your research. If you are not sure, read the appropriate sections before making your selection.

☒ Life sciences ☐ Behavioural & social sciences ☐ Ecological, evolutionary & environmental sciences

For a reference copy of the document with all sections, see nature.com/documents/nr-reporting-summary-flat.pdf

Life sciences study design

All studies must disclose on these points even when the disclosure is negative.

Sample size	No sample-size calculation was performed. Each AFM and HS-AFM experiment was done in triplicates. Fluorescence leakage assays in a duplicate. All data from succesful experiments were used for analysis. When the analysis from all multiplicates was convergent, we consider the sample size sufficient. Numbers of membrane patches, imaging areas, growth curves, and indentation curves analyzed are given in the manuscript, supporting information, or the methods section.
Data exclusions	Not all AFM and HS-AFM experiments were successfull: sometimes the membrane patches were loosely attached to mica, the liposomes did not form a supported lipid bilayer, image was lost during imaging, the AFM cantilever broke. These data were excluded from the analysis.<br>For the analysis of force-indentation curves, curves not complying with the criteria were excluded (mainly because the AFM tip was not clean during the indentation).
Replication	All AFM and HS-AFM experiments were replicated at least three times. The replicates were independent, meaning the sample (liposomes) were deposited on a freshly cleaved mica, and the membrane behavior was observed. Not all AFM and HS-AFM experiments were successfull: sometimes the membrane patches were loosely attached to mica, the liposomes did not form a supported lipid bilayer, image was lost during imaging, the AFM cantilever broke. These data were excluded from the analysis. All data from successful experiments (in at least 3 replicas) were used for analysis described in the manuscript.<br>Fluorescence leakage experiments were replicated twice, both leading to the same result.<br>Lipidomic studies were performed once. Mainly due to the expensive nature of the S. aureus lipid extract that we prepared in our lab and needed for other experiments. The lipidomics served to guide our composition of the synthetic membranes and to interpret the data. The fact that the lipidomics was done independently by two methods (mass spectroscopy and thin layer chromatography), which both gave complementary results, makes our results robust.<br>MD simulations were performed for the duration of more than 7 us, which ensures convergence of the simulated systems. All simulations lead to similar results.
Randomization	Randomization is not relevant for this study. Our study does not use groups of test subjects. Instead, we prepare membranes models of S. aureus membrane and describe the interaction of an antimicrobial with these membranes via various experimental and theoretical methods.

Blinding	Blinding was not relevant for our study. Our study does not use groups of test subjects. All analysis done is quantitative, hence, does not suffer from subjective bias.

Reporting for specific materials, systems and methods

We require information from authors about some types of materials, experimental systems and methods used in many studies. Here, indicate whether each material, system or method listed is relevant to your study. If you are not sure if a list item applies to your research, read the appropriate section before selecting a response.

Materials & experimental systems

n/a	Involved in the study
☒	☐ Antibodies
☒	☐ Eukaryotic cell lines
☒	☐ Palaeontology and archaeology
☐	☒ Animals and other organisms
☒	☐ Clinical data
☒	☐ Dual use research of concern

Methods

n/a	Involved in the study
☒	☐ ChIP-seq
☒	☐ Flow cytometry
☒	☐ MRI-based neuroimaging

Animals and other research organisms

Policy information about studies involving animals; ARRIVE guidelines recommended for reporting animal research, and Sex and Gender in Research

Laboratory animals	The study did not contain laboratory animals. The only organism used was bacteria Staphylococcus aureus, strain RN4220, NCTC8325-4 derivative, restriction deficient and cured of prophages.
Wild animals	The study did not involve wild animals.
Reporting on sex	The only organism used was bacteria Staphylococcus aureus, which does not have a defined sex.
Field-collected samples	The study does not contain samples collected in a field.
Ethics oversight	No ethical approval or guidance was required. The study contains bacteria Staphylococcus aureus, a laboratory strain not from any clinical isolate.

Note that full information on the approval of the study protocol must also be provided in the manuscript.
